# Dr. H. P. Southwick

**Published:** 1898-09

**Authors:** 


### OBITUARY.

### Dr. A. P. Southwick.

DIED, At his Residence in the City of Buffalo June 11, 1898, Alfred P. Southwick, M. D. S., aged seventy-two years.

Alfred Porter Southwick was born in Ashtabula, Ohio, May

18, 1826. The announcement of the passing away of Dr. Southwick brings a sense of personal loss, not alone to those who were intimately associated with him in life's work, but to many more who were but passing acquaintances; for the kindly nature of the man was at once apparent to all who met him. He had no enemies; for those who antagonized him could not be actuated by any feeling of honest personal resentment, or conviction of wrong-doing on his part. Those who knew him best loved him most, for his was the nature that bore the searchlight of personal intimacy. Staunch and loyal to his friends, stainlessly true in business affairs, faithful and conscientious in the discharge of every known duty, genial and kindly in his social relations, considerate, friendly and obliging with every one, it was no wonder that he was beloved by all who had opportunity to know the man as he was.

In 1853 he married Mrs. Mary M. Flinn. In 1858 he took up the study of dentistry, and in 1862, at the age of thirty-six, he gave up everything else and for the remaining thirty-six years of his life devoted himself to dental practice.

Dr. Southwick was ever a model in his professional conduct. No one ever heard him traduce or disparage a brother dentist. He was always generous, especially to younger men, and no one of them ever called on him for advice or assistance in vain. He was always prominent in professional societies, and labored earnestly to sustain them long before the present generation of dentists entered upon the scene. He was an early member of the first dental society organized in the western half of the State—The Western New York Dental Society. He took an earnest part in securing the passage of the first State dental law in 1868, and was a charter member of the State Dental Society and of the Eighth District Dental Society, within the bounds of which he resided. For twenty-one years he had been a member of the State Dental Examining Board and for fourteen years its president. In this position his duties were often very delicate and trying, yet his conduct as an officer never met with criticism at the hands of any honest man.

His reputation for intelligence and integrity, and for high professional standing, resulted in his selection for one of the most important positions when the Dental Department of the University of Buffalo was organized, and he became its first Secretary-Treasurer and Professor of Operative Technics. The chair was especially established for him, because it was believed that for this very important work he was of all men the one best fitted. The result proved the wisdom of the selection, for at the time of his death he hȧd probably brought that branch of instruction up to as high a point of efficiency as could be found in any college in the world. Without preliminary experience or training he developed into a most successful teacher. His earnestness and enthusiasm, his desire for the greatest good of others, his personal adaptation to the work and his great professional attainments, more than counterbalanced any lack of early training or discipline. His pupils loved and revered him and would apply themselves diligently that they might win his approval, when they would have been indifferent to all other inducements. He died as he had lived, solicitous for others rather than for himself, considerate and thoughtful for his associates and those whom he loved, rather than anxious on his own account. Without ostentatious professions he passed away, the true Christian, who came nearest to that highest type of human development, the man who dealt with his neighbor as he would have others deal with him.

